# Supplementary material for: Multi-Stimulus Responsive Multilayer Coating for Treatment of Device-Associated Infections
Source: J Funct Biomater. 2022 Feb 28;13(1):24. doi: 10.3390/jfb13010024 (PMC8954600; doi:10.3390/jfb13010024)
Supplement: Supplementary file 1 [file jfb-13-00024-s001.zip › jfb-1598459-supplementary.pdf]

Supporting Information

Multistimulus-Responsive Multilayer Coating for Treatment of Device-Associated Infections

Wenlong Li[1], Guanping Hua[1], Jingfeng Cai[1], Yaming Zhou[1], Xi Zhou[1], Miao Wang[1*], Xiumin Wang[4] , Baoqing Fu[3*], Lei Ren[1 2*]

1 The Higher Educational Key Laboratory for Biomedical Engineering of Fujian Province, Research Center of Biomedical Engineering of Xiamen, Department of Biomaterials, College of Materials, Xiamen University, 422 Siming Nan Road, Xiamen 361005, P. R. China.; 183850396@qq.com (W.L. Li), 1049750351@qq.com (G.P. Hua), 1215591273@qq.com (J.F. Cai), 515917328@qq.com (Y.M. Zhou), xizhou@xmu.edu.cn (X. Zhou), mi-aowang@xmu.edu.cn (M.W. Wang), renlei@xmu.edu.cn (L. Ren)

2 State Key Laboratory of Physical Chemistry of Solid Surfaces, College of Chemistry and Chemical Engineering, Xiamen University, Xiamen 361005, P. R. China.; renlei@xmu.edu.cn (L. Ren)

3 Department of Laboratory Medicine, Xiang'an Hospital of Xiamen University, School of Medicine, Xiamen University, Xiamen 361102, P. R. China. 1350750090@qq.com (B.Q. Fu)

4 School of Pharmaceutical Sciences, Xiamen University, Xiamen, 361102, P. R. China. wangxm@xmu.edu.cn (X.M. Wang)

* Correspondence: E-mail addresses: renlei@xmu.edu.cn (L. Ren), miaowang@xmu.edu.cn (M. Wang), 1350750090@qq.com (B.Q. Fu).

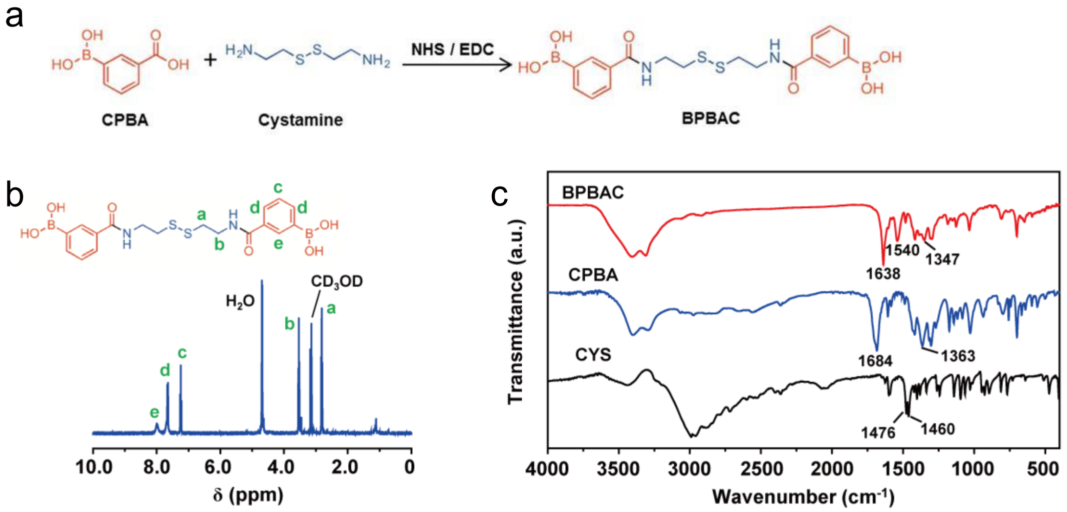

Figure S1. (a) Chemical structural formula of BPBAC; (b) ^{1}H-NMR spectrums of BPBAC; (c) FT-IR absorbance spectrums of CPBA. CYS, and BPBAC.

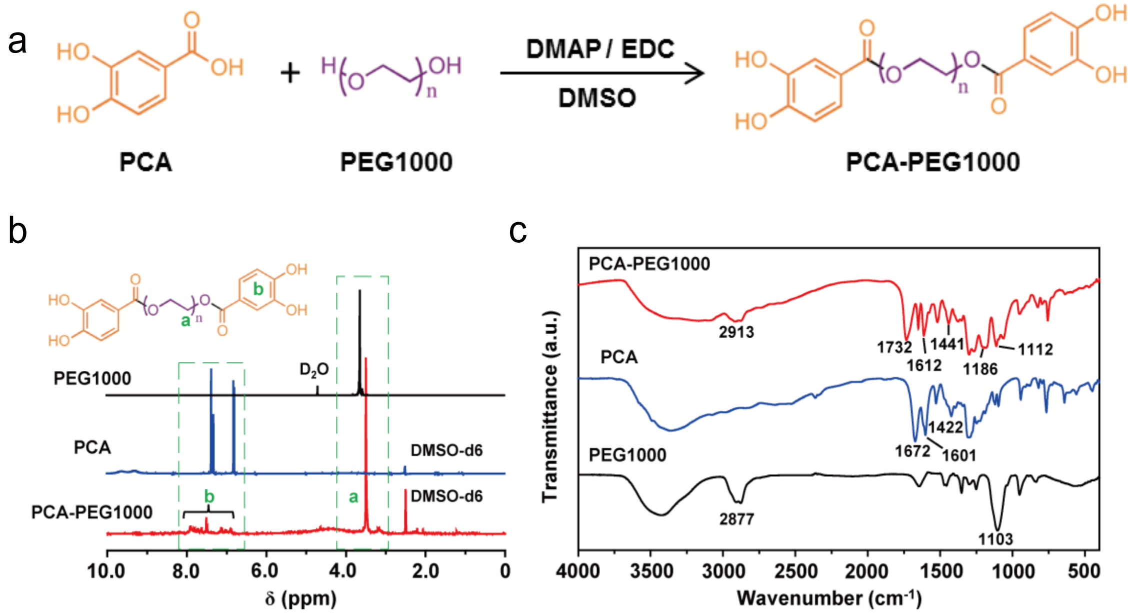

Figure S2. (a) Chemical structural formula of PCA-PEG1000; (b) ^{1}H-NMR spectrums of PEG100, PCA, and PCA-PEG100; (c) FT-IR absorbance spectrums of PEG100, PCA, and PCA-PEG100.

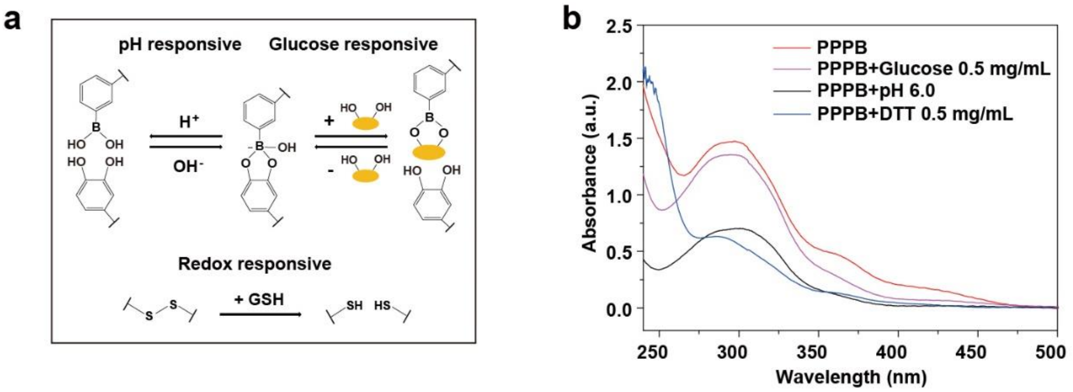

Figure S3 a) The responsive mechanism of PPPB; (b) UV absorption spectrums of PPPB in PBS buffer (pH 6.0), glucose aqueous solution (0.5 mg/mL) and DTT aqueous solution (0.5 mg/mL).

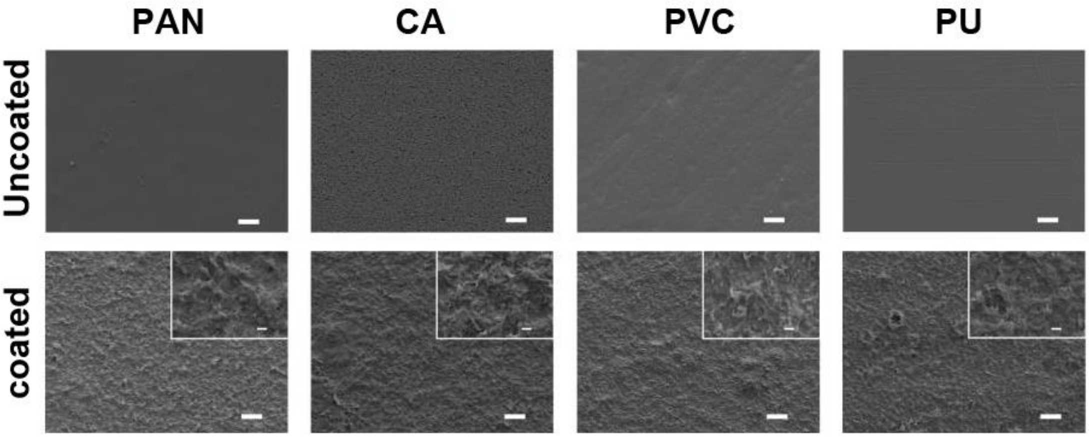

Figure S4 SEM images of the surfaces of CA, PAN, PVC and PU substrates coated and uncoated with $(MMT\text{-}PPPB\text{-}CHA)_3$. Scale bar: 2 μm.

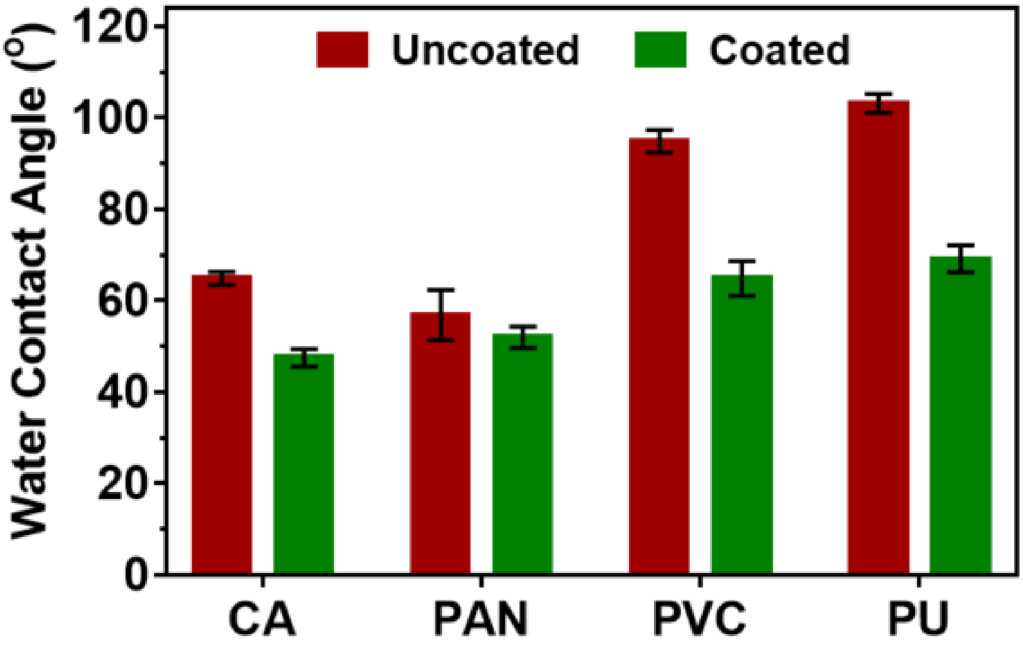

Figure S5 Water contact angles on the surfaces of CA, PAN, PVC and PU substrates uncoated and coated with (MMT-PPPB-CHA)$_3$.

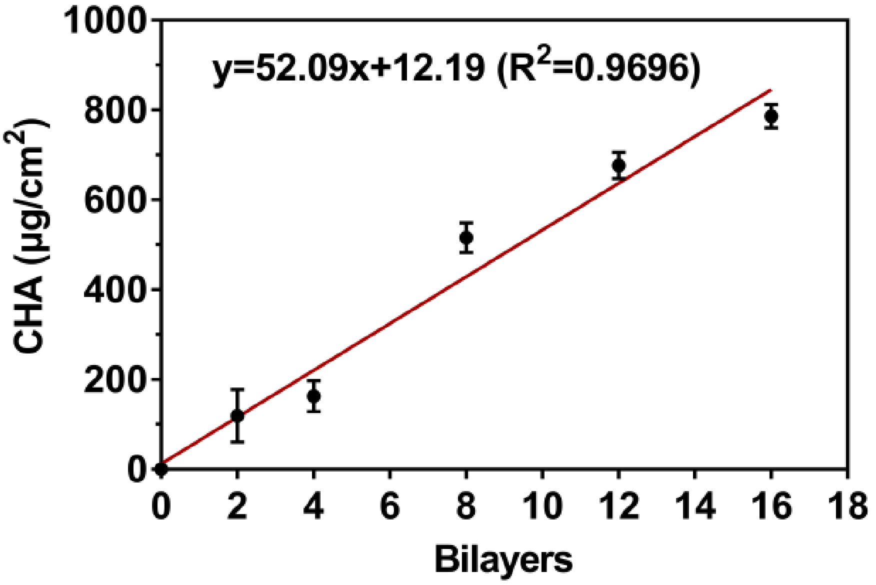

Figure S6 The relationship between drug loading and the number the (MMT-PPPB-CHA)$_3$ bilayers

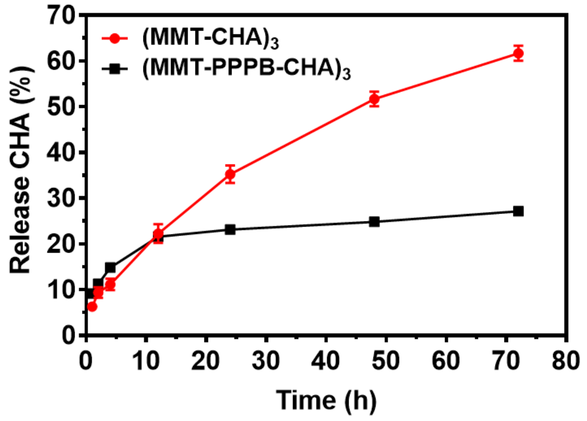

Figure S7. CHA release curves of $(MMT-CHA)_3$ and $(MMT-PPPB-CHA)_3$ coating in 72 hours.

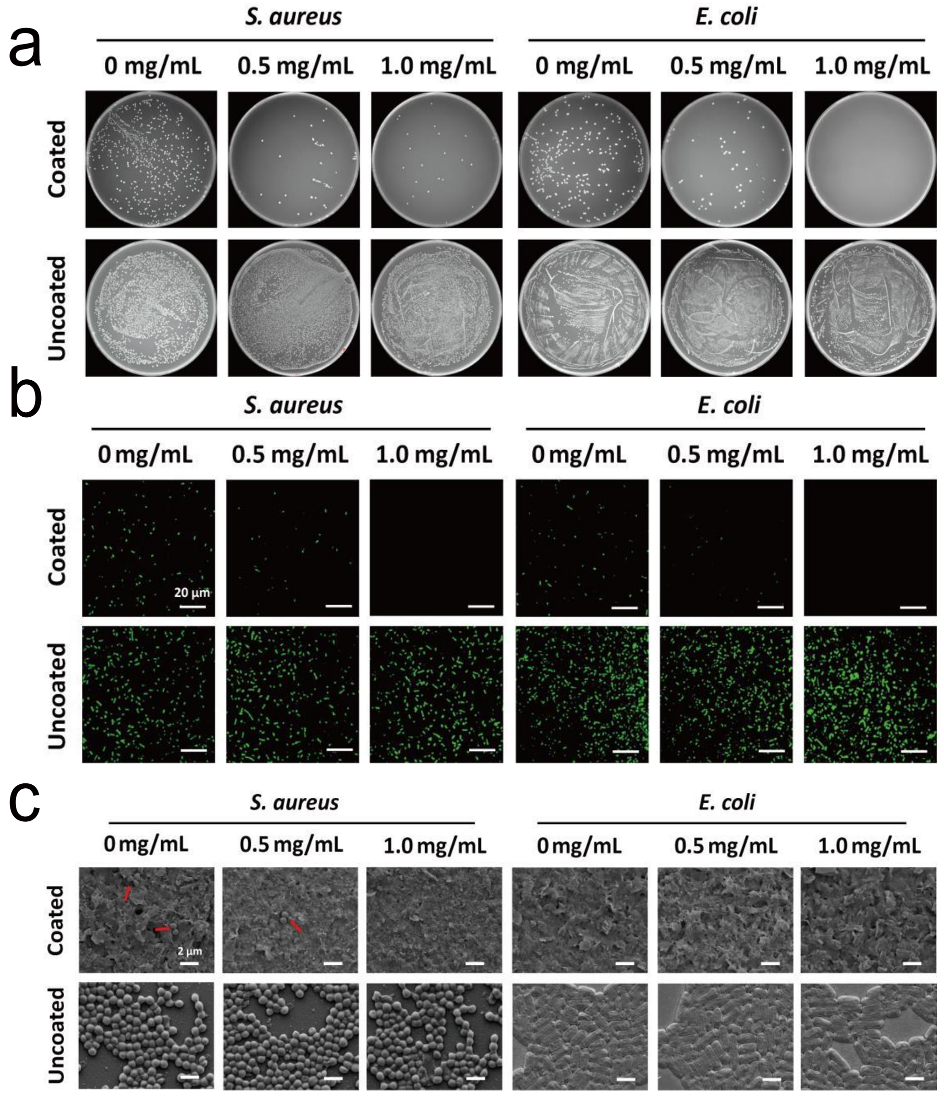

Figure S8 (a) Images of bacterial clones on the agar plates after treatment with (MMT-

PPPB-CHA)3 coating for 2 h in glucose solutions (0, 0.5, 1.0 mg/mL); (b) CLSM diagrams and (c) SEM images of bacterial adhesion on the (MMT-PPPB-CHA)3 coating for 2 h in glucose solutions (0, 0.5, 1.0 mg/mL).

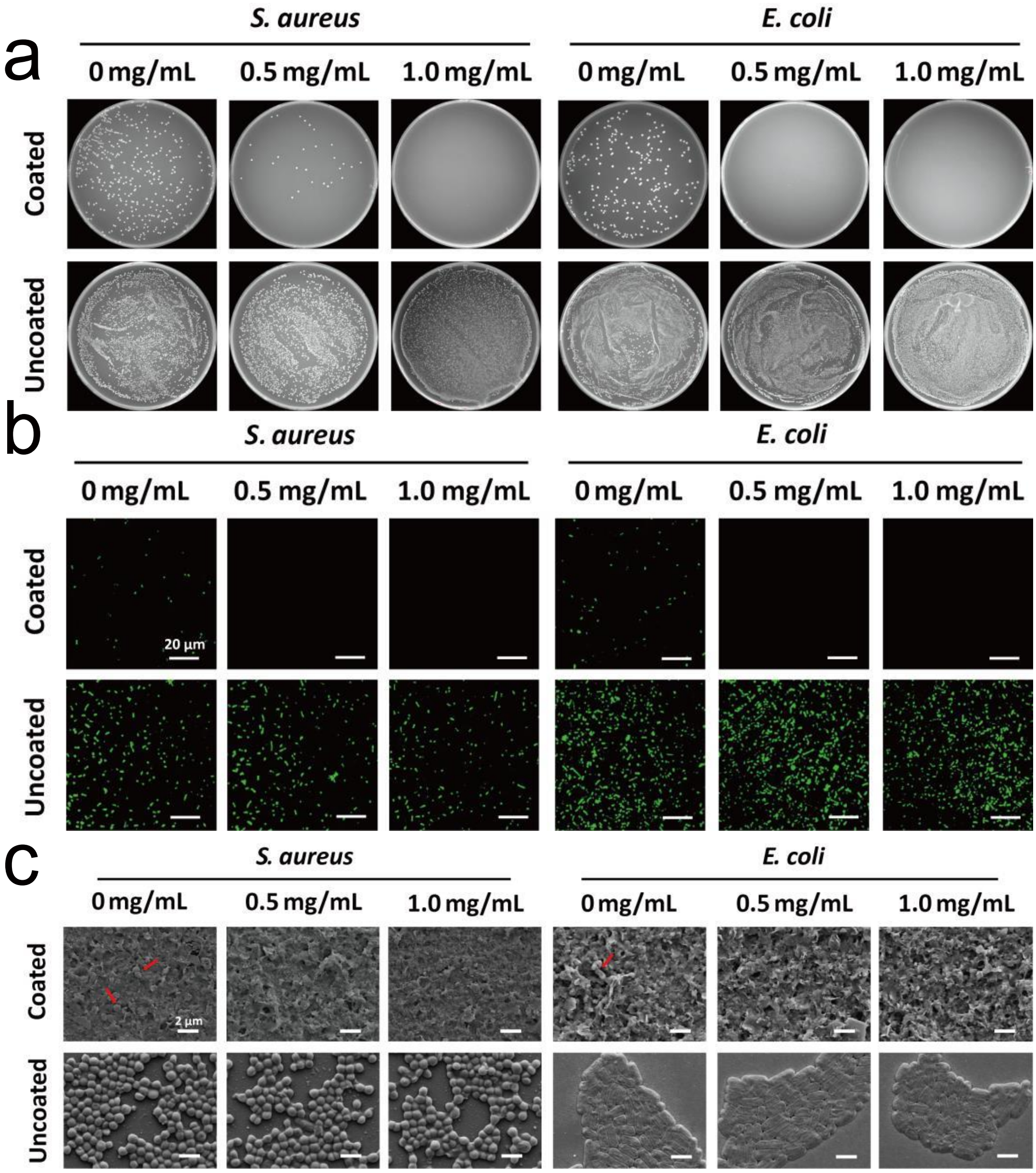

Figure S9 (a) Images of bacterial clones on the agar plates after treatment with (MMT-PPPB-CHA)3 coating for 2 h in DTT solutions (0, 0.5, 1.0 mg/mL); (b) CLSM diagrams and (c) SEM images of bacterial adhesion on the (MMT-PPPB-CHA)3 coating for 2 h in DTT solutions (0, 0.5, 1.0 mg/mL).

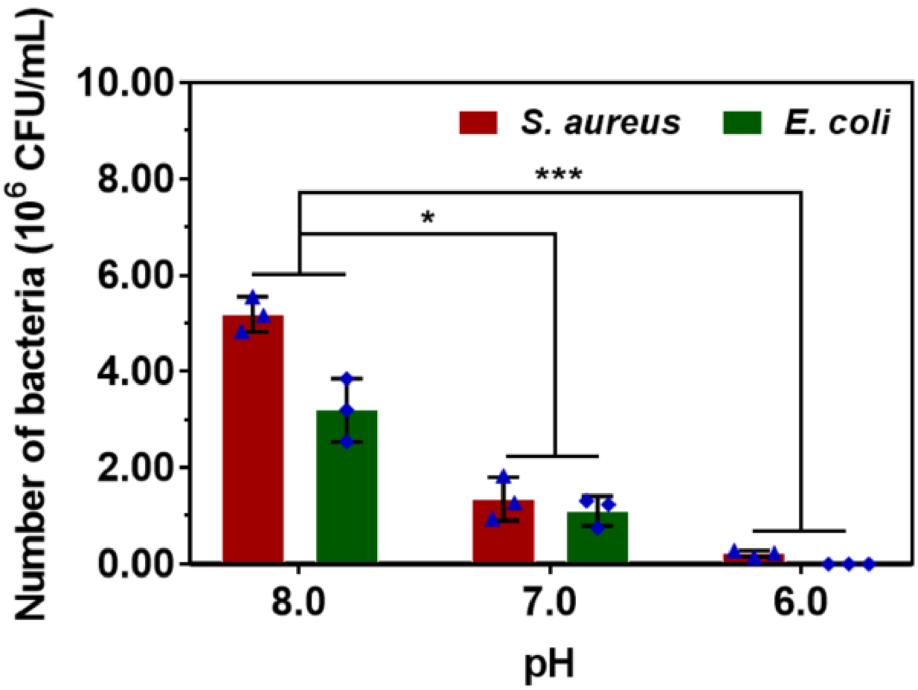

Figure S10 Concentrations of *S. aureus* and *E. coli* after incubating with (MMT-PPPB-CHA)$_3$ at different pH values (6.0, 7.0, 8.0) for 2 h. *$p < 0.05$, ***$p < 0.001$

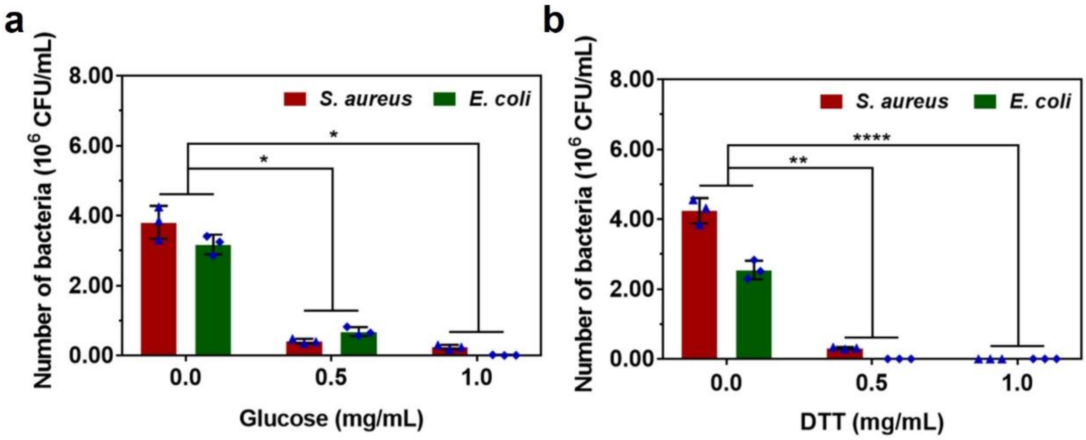

Figure S11 Concentrations of *S. aureus* and *E. coli* after incubating with (MMT-PPPB-CHA)$_3$ under different (a) glucose and (b) DTT concentrations (0, 0.5, 1.0 mg/mL) for 2 h. *$p < 0.05$, **$p < 0.01$, ***$p < 0.001$.

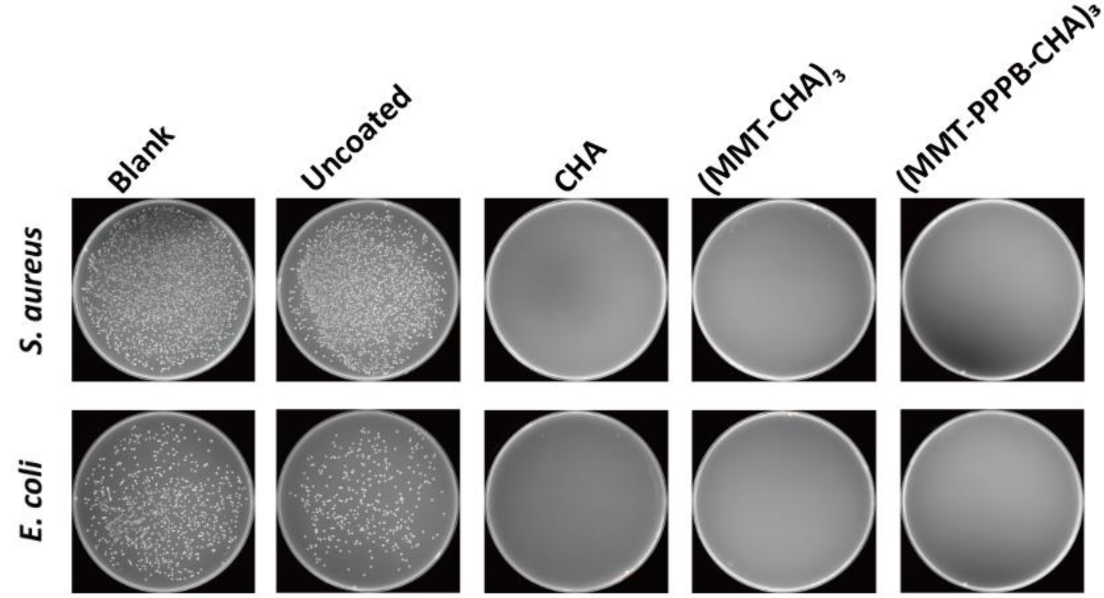

Figure S12 The growth of *S. aureus* and *E. coli* in infected wound tissues after 14 days of different treatments.
